# Midwifery

**Published:** 1824-06-01

**Authors:** 


### Midwifery.

1. *Constipation in Pregnancy.* Nothing is more uncertain than the effect of constipation. One person will be in perfect health with only a single alvine evacuation weekly; while another will be quite unhinged if he pass many hours beyond the accustomed period of visiting the "Mont de Piete." Dr. Lemazurier has lately published a case, the prominent particulars of which we shall lay before our readers. He was sent for on the 8th July 1823, to see a woman in the seventh month of her third pregnancy. She had been of a costive habit from youth, and had abused the use of purgatives. For the three last months she might be said to have had no passage through her bowels—notwithstanding a milk and cooling diet. She had no fever—the tongue a little discoloured—the pulse and appetite feeble. After taking any solid food she was troubled with dyspnœa and general malaise. The abdomen was much distended—the motions of the fœtus very distinct—the transverse arch of the colon was felt greatly distended with an immense accumulation of fæces. The ascending colon and sigmoid flexure were in a similar state. There was no pain on pressure of the abdomen. This state of things caused dyspnœa, anxiety, sleeplessness, tendency to faintness, and pains in the loins. She had been bled before our author's visit. He therefore confined his prescriptions to milk diet, rigid abstinence from solid food, diluents, lavements, semicupia. These, as might be expected, proved totally useless; and it was determined to wait till the accouchement was over, before the evacuation of the bowels was attempted! This event took place on the 11th of September, and a well formed child was delivered without much trouble. The situation of the colon immediately changed, and took up a position near the pelvis. Symptoms of abdominal inflammation and irritation now came on, in addition to milk fever, which was developed on the 14th of the same month. Linseed tea, fomentations, leeches, and the warm bath, did not effect much. Nevertheless, by means of glysters, and other very gentle measures, the fæcal accumulation appeared to break up, and there was an evacuation of two or three pounds of hard, brown, and fetid matters. But there remained a collection too large for expulsion. The unhappy patient was worn down by nausea, fever, colicky pains, meteorism of the abdomen, retention of urine, procidentia vesicæ; and expired on the 21st of September.

*Dissection.* We shall pass over a number of minute particulars, in order to give only the essential pathological characters. The peritoneum was inflamed throughout its whole extent. There was a sero-purulent effusion in the cavity of the abdomen—false membranes over various portions of the intestines—some inflammation of the mucous membrane of the small intestines. The colon, from the cæcum to the rectum, was intensely inflamed, and its volume enlarged to a frightful extent. It was a foot in circumference, throughout the whole or nearly the whole of its length. It was filled with gas, and with $13\frac{1}{2}$ pounds (French) of solid fæces!

It is doubtful whether any means would have been successful at the time Dr. Lemazurier visited the patient; but we question the propriety of waiting two months for the delivery of a child before an attempt was made to open the bowels of the mother. Surely, if the life of a parent be more valuable than that of a fœtus in utero, it would have been perfectly justifiable to procure premature delivery rather than allow fæces to accumulate for two additional months in the colon. When the physician was called in, this measure unquestionably presented more chance of saving the patient's life than the measure pursued by the Medical Adviser.

---

2. *Rupture of the Fallopian Tube.* In the process of utero-gestation the quantity of blood supplied to the parts concerned, and the activity of the vessels there must be prodigious. Rupture of a very small vessel about the ovaries or fallopian tubes, in the early weeks of pregnancy, will cause immense and fatal hæmorrhage in a very short time.

Mr. Bushell, of Crawford Street, has lately published a case of this kind in our cotemporary, the Medical and Physical Journal, of which we shall take a short notice. At 11 o'clock at night a pregnant female was suddenly seized with violent pain in the abdomen, and fainted away. On recovering a little she was placed in bed and appeared to be dying, in which state she continued till the morning. A practitioner had been sent for, but declined visiting the poor woman! We wish Mr. Bushell had given the name, for surely this was a most flagrant dereliction of duty. At 5 o'clock in the morning Mr. Bushell found the patient with hands and feet quite pale and cold—eyes glassy—great restlessness, but perfectly sensible. She could not bear the least pressure on the abdomen. Fomentations externally—stimulants internally. At 10, A. M. nearly in the same state. She expired about 12 o'clock.

This poor woman was of a lively and cheerful disposition naturally; but her husband's health and affairs being embarrassed, she stifled her griefs, while she consoled and supported the drooping spirits of her partner.

*Dissection.* Abdomen slightly tumid. When opened, an abundant flow of bloody serum now took place, to the amount of three pints at least. On turning up the intestines, a large mass of coagulum, about two pounds, was found in the pelvis. The aorta, vena cava, and other abdominal blood-vessels, being examined, no rupture could be found. The uterus was but slightly enlarged—not more so than in the first month of pregnancy. Os uteri blocked up with viscid mucus. The internal surface of the organ was turgid, with a muco-flocculent lining, the incipient decidual membrane; there was also an increase of vascularity at each tubal aperture. The left fallopian tube and appurtenances were healthy, as was the *right*, for three-fourths of its fimbrial extremity. On inflation it was pervious, and had the appearance as if an ovum had very recently passed through it. The uterine termination was enlarged and nearly twice the size of the left fallopian tube at the same part. On the posterior aspect of this enlargement, a small, irregular, jagged hole was visible, which would barely admit the extremity of a probe. On tracing the tube towards the uterus, a vesicle, about half the size of a pea was discovered, and proved to be the ovum. The minute embryo, not larger than a small pearl barley, was perfectly distinct, floating in its liquor amnii. For several more minute particulars we refer to the original paper. We saw the recent preparation, and can vouch for the fidelity of Mr. Bushell's detail.

---

3. *Sloughing of the Bladder after Parturition.* As this is one of the most deplorable accidents which can follow delivery, and is rarely cured, it is desirable to give every publicity to successful issues. Mr. James Guthrie, of Kilmarnock, has lately recorded an instance of this kind.

Mrs. Campbell, aged 23, had been three days in labour, of a first child, before delivery was effected—and that ultimately by means of the forceps. In the early stages of parturition she made water frequently, but towards the end, a total retention took place, nor could the catheter be introduced. On visiting the patient next day, she complained of an excessive discharge from the vagina, with pain and tenderness of the external parts. She had discharged her urine about an hour after delivery, but had no power of retaining it since. Symptoms of an inflammatory nature now came on, requiring decided depletion, and when these symptoms subsided the melancholy fact of sloughing of the bladder was unequivocally ascertained. The situation of the aperture was about, or rather above the cervix vesicæ—its edges felt soft and irregular—and were painful to the touch. A piece of sponge was introduced into the vagina, as soon as the tenderness of the parts would admit, and applied in direct contact with the perforation in the bladder. A short elastic gum catheter was next passed into the urethra, and being fixed by suitable bandages, was allowed to remain there, in order to prevent any accumulation of urine in the bladder. In this way the edges of the aperture were brought into more immediate approximation. The report three days afterwards was—"the urine flows entirely by the catheter; sponge completely prevents it from passing into the vagina; feels much more comfortable; considerable discharge of offensive matter from the vagina." Two days subsequently there was "excessive discharge of fetid matter from the vagina, with great pain and tenderness of the external parts, which are now in a state of ulceration—urethra very irritable. The sponge and catheter withdrawn, cleaned, and again introduced. In three days more the pain and tenderness so great that the sponge and catheter were again withdrawn by the patient herself. At this time an examination was made *per vaginam*, and the original aperture was found so greatly diminished, as scarcely to admit the point of the finger. With much persuasion, she again submitted to the introduction of the sponge and catheter, which, in three days more, were withdrawn to be cleaned. They were again replaced. The above treatment was continued, with little variation, for a month, at the end of which time, the aperture in the bladder was completely shut up, by a soft but pretty firm cicatrix. The communication was thus entirely obliterated. She was examined five months afterwards, and found perfectly free from complaint.

Mr. Guthrie is entitled to much credit for his perseverance in removing an affliction which renders life a horrible and loathsome evil, instead of a blessing and enjoyment.

---

4. *Self-performed Cæsarean Section.* Some marvellous cases of this kind are on record—but though on record, are of very suspicious authenticity. Dr. Moseley has related the case of a negro woman in Jamaica, who performed the Cæsarean operation on herself, by cutting boldly through the uterus, and extracting a child from the left side of the abdomen. This operation was performed with a butcher's knife. The child died of lock-jaw, but the mother recovered. Extraordinary and incredible as this case may appear, it is more than equalled by a case recently published in our respected transatlantic cotemporary, the New York Medical and Physical Journal, for March 1823. It is reported on the authority of more than one or two medical men. The *operator* was a young servant girl, " one fourth black," and only 14 years of age! while the family were at dinner, she went a little way from the house, and placed herself on a wreath of snow, where she was discovered by her master in the act of covering something with snow—which proved afterwards to be a naked child. As soon as perceived " she immediately ran to the house with the second child hanging out at the wound, together with a considerable portion of her intestines." She was now surrounded by two medical men, Dr. Basset and another. A wound was found near the centre of the epigastric region, from which the second fœtus was extracted. This wound was four inches in length, extending in a diagonal direction, as respected the abdomen, about two inches above the umbilicus, with another incision, at nearly a right angle with the former, extending toward the sternum. The lower part of the abdomen was considerably distended with blood. This was first evacuated by changes of posture and gentle compression. The wound was then sewed up, and a bandage applied. She recovered. " I should judge, says the reporter, from the appearance of the blood upon the snow (there being three several places where she evidently stopped,) that the incision was made *immediately* preceding the rupture of the membranes, and that the first child was delivered *per vias naturales*, the third pain after the rupture."

The above case was reported to the Rennselaer Medical Society by Dr. Samuel M'Clellan, (who appears to have been the other physician in attendance) the president of the society, and by them forwarded to several of the American Journals. It is one of those extraordinary cases which we cannot easily believe, yet dare not positively deny.

---

5. *Pressure on the Perineum.* We some time ago threw out a hint of our scepticism respecting the efficacy of pressure on the perineum, as a preventive of rupture of that part in parturition. Dr. Campbell, of Edinburgh, has taken us to task on that point, but we urged our opinions no farther. We now see in the same journal through which Dr. Campbell's animadversions were made, a short paper from the pen of Mr. Thompson of Whitehaven, in which he seems to agree with us, that pressure on the perineum, in parturition, is more likely to cause than prevent laceration of that part. "It is possible that the force used may facilitate what it is meant to prevent; and the perineum, instead of being saved, be eventually torn." We certainly think it is reasonable to expect that, when a substance, soft in comparison, is placed between two bodies strongly opposed to each other, if there be a disposition to give way, it is the softest body that is likely to suffer. Our author strongly recommends the plan of Dr. Barlow (a gentleman of great experience) which is, to keep back the head, when it is advancing too rapidly, by the fingers applied to the head itself, and not to the perineum.—*Ed. Journal.*

---

6. *Sudden Parturition.* Every accoucheur in extensive practice must have met with cases of remarkably quick parturition. These cases are chiefly interesting in a forensic point of view, as the plea of sudden birth has been sometimes urged in behalf of an unfortunate mother accused of destroying her illegitimate offspring. Instances of this kind, well authenticated, should therefore be put on record, for the aid of medical witnesses. Mr. Tatham *(Med. Repos. April)* has related two cases of unusually rapid parturition. One was a clergyman's wife, who, while sitting in her parlour, the rest of the family being at chapel, was taken ill (in the 9th month of utero-gestation) and obliged to go to the night chair. A great discharge of water took place, followed immediately by twin children, which dropped into the utensil. An alarm was given, assistance obtained, and the children rescued from their cold watery bed. They died within the week.

The other case was nearly similar. A woman felt three trifling pains, while sitting below stairs, but did not complain. In her passage to the bed room a child was suddenly expelled, and fell on the floor of the lobby, bleeding profusely from the umbilical cord. By prompt assistance, Mr. Tatham saved both mother and child. In the first case it was the lady's first child—in the second, it was the fourth pregnancy.

